# From dividing to dormant: embracing the full activity spectrum for environmental microorganisms

**DOI:** 10.1128/mmbr.00354-25

**Published:** 2026-06-25

**Authors:** Ludivine Guigard, Victor Bal, A. Fina Bintarti, Margaux Buron, Emma Chavan, Milena Gonzalo, Xipeng Liu, Wei Zheng, Ashley Shade

**Affiliations:** 1 Université Lyon 1, CNRS, INRAE, LEM, UMR 5557 27098, Villeurbanne, France; 2 College of Resources and Environment, Northwest A&F University, South Campus of Northwest A&F University 12469, Yangling, Shaanxi, China; Universite de Lausanne, Lausanne, Switzerland

**Keywords:** dormancy, VBNC, structure-activity relationships, community size, active taxa, active cells, persister, spore, storage effect, climate change

## Abstract

Microorganisms can cope with stress by entering dormancy, a viable state of reduced metabolic activity that enables persistence, dispersal, and long-term survival. However, microbial life in environmental systems is best understood as a spectrum of metabolic activity, spanning from highly active, dividing cells to deeply dormant phenotypes. This spectrum reflects dynamic survival strategies under fluctuating conditions, with critical implications for ecosystem stability, gene dissemination, and resilience to disturbances in natural and human-influenced systems. Yet, microbial activity is often treated as binary (active vs. dormant), oversimplifying a biological continuity that remains technically difficult to quantify. Here, we synthesize advances in microbial dormancy to reconceptualize activity as a spectrum. We review current and emerging methods to quantify environmental activity, linking each to the Central Dogma of molecular biology (DNA to RNA to protein) to guide interpretation along a generalizable continuum. Through a literature synthesis of terrestrial, aquatic, and wastewater treatment ecosystems, we compare how methods estimate active cells and populations. We recommend standardized reporting of total community size, active cell abundance, and proportional activity to enrich the interpretation of microbiome ‘omics data, with activity intensity and active-inactive switching providing deeper insights. To achieve this, we advocate for increased accessibility and throughput of precise activity-discriminating technologies, alongside renewed use of reliable methods like direct cell counts and activity stains. Adopting this spectrum-based perspective will improve our ability to tackle key societal challenges, such as understanding microbial contributions to ecosystem function under climate change and gene dispersal at human-environment interfaces.

## INTRODUCTION

Microorganisms living in the environment often face harsh, fluctuating, and resource-limited conditions. They have evolved strategies to reduce metabolic activity, self-protect, and persist, with the extreme case being a state of minimum metabolic activity called dormancy (1–3). Dormant microbes can switch back into a reactivated state, and there exhibit a range of activity intensities, with dividing cells at the maximum extreme. As a trade-off for protection, the individual cost of reduced activity is the temporary inability to reproduce. Thus, the capacity for deactivation and dormancy is a critical factor that underlies microbial success: it protects cells as they disperse (4), sustains microbial biodiversity (5, 6), and provides a biological memory of adaptations to past conditions (7).

Despite the importance of dormancy for survival and persistence, the phenotypic state of viable minimum activity for most environmental bacteria is unknown. The exceptions are a few well-studied models, including endospore-forming bacilli, exospore-forming Actinomycetota, cyst-forming Cyanobacteria, and cyst-forming *Azotobacter* (8, 9). This dearth of basic biological knowledge hinders our fundamental understanding of environmental microbiology. For example, soils contain the highest bacterial diversity known, with tens of thousands of taxa (e.g., reference 10), 10^29^ cells inhabiting the terrestrial biosphere (11), and a previously reported average of 80% inactive cells (1). Thus, most of terrestrial bacterial life persists in a low-activity state about which there is little knowledge. While soil may be an extreme in its expected degree of microbial inactivation, this lack of knowledge about microbial dormancy applies generally to most environmental systems.

Importantly, the ability of inactive environmental microbes to reactivate at varying intensities and thereby shift community functions has major implications for environmental and human health. This includes ensuring the stability of ecosystem functions after disturbances and facilitating the dissemination of genes across environments, particularly in human-managed systems like wastewater treatment and food production (e.g., references 12, 13). Reactivation can unlock previously inaccessible functions and genetic traits within microbial communities. For instance, environmental bacteria carrying antibiotic resistance genes on mobile genetic elements may spread these genes after reactivating (14). Similarly, reactivation can activate or repress genetic pathways involved in carbon and other nutrient cycling, influence climate-critical ecosystem services and our ability to predict them (15–17, 18, 19), and enhance bioremediation potential (20). Additionally, some low-activity cells, such as antibiotic-tolerant persisters, play a key role in infectious diseases, contributing to hard-to-treat infections (21–23).

Our thesis is that treating microbial activity as a quantifiable continuum is essential for mechanistic inference in environmental microbiology and for addressing major societal challenges, such as One Health, sustainable agriculture, climate change, and microbial conservation. We first define an activity spectrum spanning from dividing cells to deep dormancy, distinguishing binary *activity states* from quantitative *activity levels* anchored to core molecular processes. We then synthesize recent advances in dormancy microbiology within this spectrum framework and summarize key ecological consequences for populations and communities, and their contributions to ecosystem functioning. Next, we evaluate current methods for discriminating environmental microbial activity states and levels, and we use a cross-system literature synthesis to summarize reported contributions of active cells and populations across terrestrial, aquatic, and wastewater treatment environments. We conclude with priority knowledge gaps and practical reporting recommendations to support quantitative activity measurements in environmental microbiology.

## THE MICROBIAL ACTIVITY SPECTRUM

**Fig 1:**
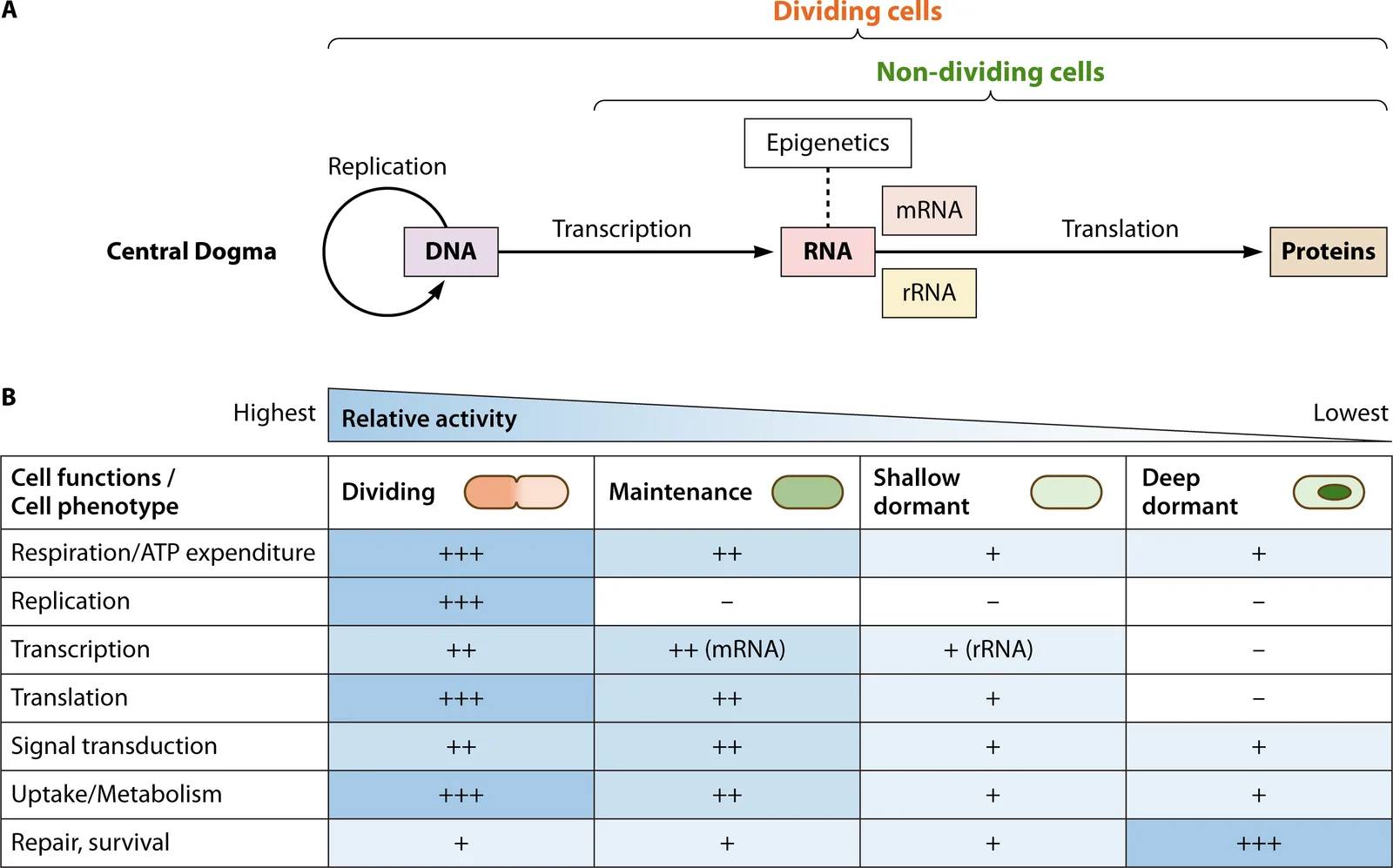
The activity spectrum of microorganisms is linked to cellular processes and the Central Dogma. (**A**) Representation of the Central Dogma (DNA to RNA to protein) as well as dividing (i.e., orange) and non-dividing (i.e., green) cell alignment to these processes. (**B**) Representation of the activity spectrum depending on a cell’s level of activity and cellular processes. This spectrum is nested in that the most basal activities will also be observed in cells that have higher activity (e.g., dormant cells may retain limited respiratory activity, whereas DNA synthesis is restricted to actively replicating cells). The symbols “+++,” “++,” “+,” or “-” and the color intensity in the table represent a cell’s expected investment in that function relative to others at a given activity level along the spectrum.

While there is no consensus definition of microbial dormancy (3), most microbiologists would agree that dividing cells are active and that sporulated microorganisms are dormant. Yet, an activity spectrum exists between these extremes (Fig. 1). A thoughtful conceptualization of an activity spectrum was previously presented by Blagodatskaya and Kuzyakov (24) to describe the expected timing of soil microbial community reactivation, especially after substrate addition, with the continuum encompassing dead to dormant to potentially active to active. Another dormancy continuum hypothesis was proposed to describe the relationship between viable but non-culturable (VBNC) and persister cells facing antibiotics (e.g., references 25, 26), though this continues to be debated, with some arguing that VBNC cells are misclassified dead cells (e.g., reference 27). Still, a default binary definition of either active or dormant has persisted as a false dichotomy, potentially due to the intensive study of model microorganisms with specialized dormant phenotypes as well as methodological limitations in observing gradations of activity. For those microbes with specialized dormant phenotypes (e.g., endospore-forming bacilli [28] and cyanobacteria under starvation [29]), the cell commits to dormancy by investing in physical restructuring, which is also associated with characteristic metabolic shifts. However, these phenotypes may be exceptions rather than the rule.

There is mounting evidence for an activity spectrum from dividing to dormant in many microorganisms. For example, a dormancy depth was observed in *Escherichia coli* persister cells and quantified by protein aggregation within the cell and by ATP levels, with larger aggresomes and lower ATP levels associated with deeper dormancy (30). Furthermore, the depth of dormancy was dynamic and reversible, depending on ATP availability (23, 30). A dormancy spectrum was also suggested for yeasts maintaining activity at low temperatures (31, 32). Furthermore, there are numerous examples of metabolically active but non-dividing bacterial cells. This includes subpopulations of starved bacilli that, instead of sporulating, could remain in an “oligotrophic growth state” that is transcriptionally distinct (33), stationary-phase bacteria consortia that continue to invest in both interference and exploitative competition (34), and soil microorganisms that use maintenance energy to survive low water stress by oxidizing trace gases like hydrogen gas (35, 36). Some metabolically active, non-dividing cells appear to be preparing for future growth by reprogramming their transcriptional pathways (e.g., reference 37; also discussed for *Mycobacterium tuberculosis* in reference 2). Other non-growing cells are preparing for or recovering from stress, with functions for repair upregulated (38, 39). Collectively, these studies and others strongly support a reconceptualization of microbial activity from a binary state to a spectrum.

To generalize the full spectrum of environmental microbial activity from dividing to deeply dormant cells, universal cellular processes can be used as reference points to capture differences in activity levels and states. Specifically, the Central Dogma of molecular biology, in which information from DNA is transcribed to RNA, which is translated to protein, forms the basis (Fig. 1).

The use of binary activity thresholds is most defensible at the two extremes of the activity spectrum: division and deep dormancy (e.g., sporulation). Dividing cells are actively replicating their DNA and typically show the highest levels of transcription/translation and ATP demand as they build progeny cells. In contrast, spores (and other protective resting phenotypes) are deeply dormant: DNA replication is halted, transcription and translation are largely paused, and only minimal metabolism persists (e.g., basal levels of respiration and ATP expenditure for maintenance and repair). Thus, division and deep dormancy provide clear endpoints where binary classification is most appropriate.

In between these extremes are metabolically active but non-dividing cells. These cells interact with their environment while maintaining lower and variable levels of transcription, translation, respiration, and ATP expenditure. They are neither replicating nor deeply dormant, but occupy intermediate, dynamic positions along the activity spectrum. Therefore, methods that quantify the intensity of core processes (transcription, translation, respiration, and ATP expenditure) are best suited to resolve activity along the spectrum. Linking methods to the processes they measure provides a common interpretive framework and can improve cross-study comparability.

With these common reference points, activity can be compared across habitats in standardized terms, even when absolute activity differs. For example, an Arctic permafrost microbial community may be less active than a temperate rhizosphere community in an absolute measurement, but its standardized activity changes can be compared to understand characteristic dynamics and responses to perturbation relative to the rhizosphere community. This allows for improved understanding of sensitivity and recovery among communities inhabiting different environments.

Simple, categorical definitions can be applied along the activity spectrum without losing their intention to bring awareness to the biological continuum. For example, deep dormancy can be used to refer to apparent phenotypes that require more energy and time to achieve a dividing state. Shallow dormancy can refer to reduced volume cells or similar protective phenotypes and can more quickly achieve growth than deep-dormant cells. Maintaining cells are those that are most primed for division, should conditions shift favorably. These categories should reflect an expected trade-off in protection versus cell responsiveness to a growth-favorable environment, but, for many non-model environmental microorganisms, these trade-offs have yet to be studied.

## RECENT ADVANCES IN UNDERSTANDING DORMANCY AND ACTIVITY

Given that several recent reviews have discussed mechanisms that drive microbial dormancy and awakening (e.g., references 21, 40, 41), here we provide a brief overview, focusing on recent advances and linking to broader generalities where possible. The cellular mechanisms underpinning switching along the activity continuum are, of course, directly linked to the Central Dogma: halting or enhancing DNA replication, transcription, or translation, and their various regulatory mechanisms. These form the basis of both activity reduction and an increase in the microbial world.

### Inactivation

**Fig 2:**
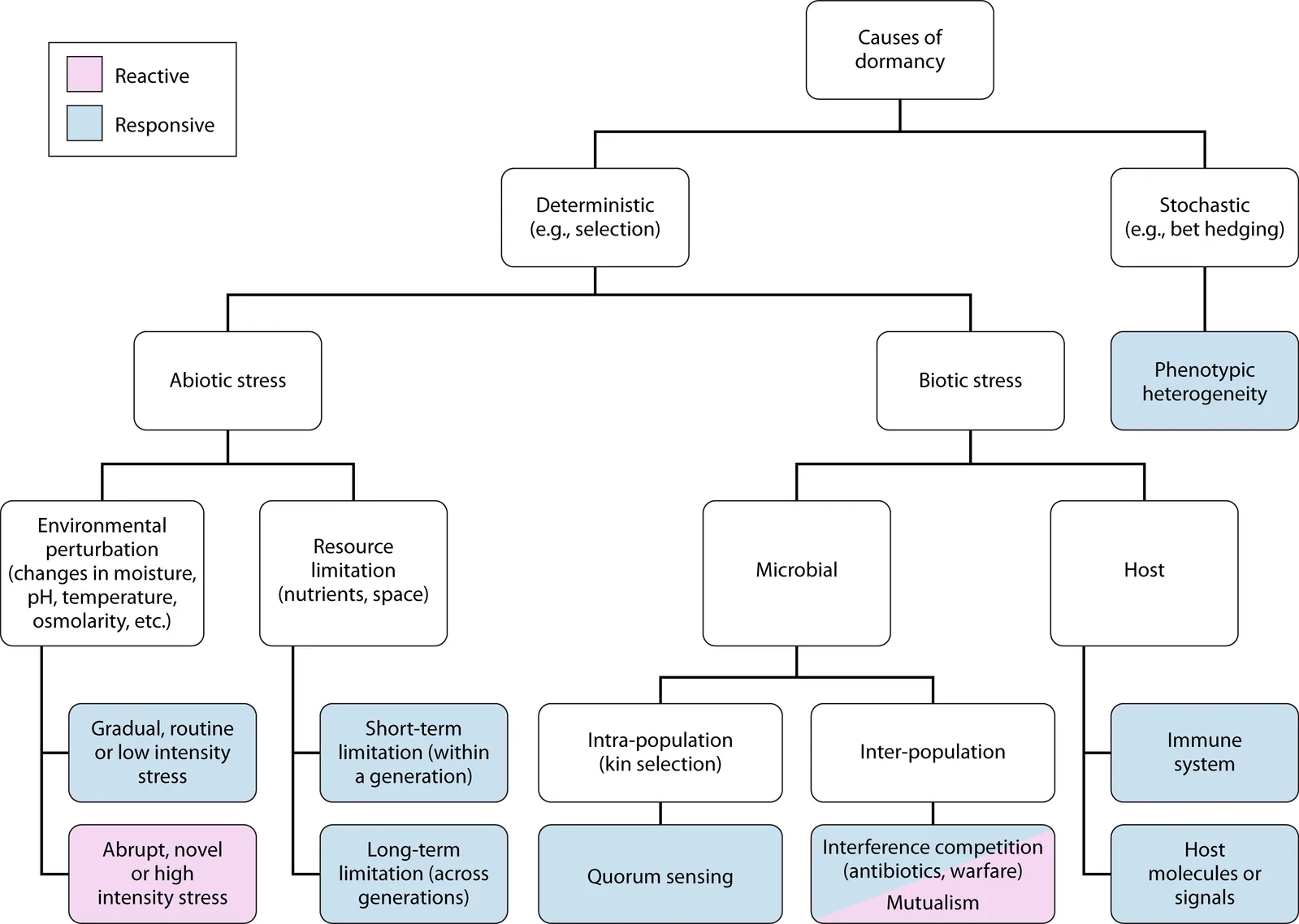
A proposed framework to categorize the causes of microbial initiation into dormancy. Reactive dormancy initiation examples are provided in pink (e.g., in response to an abrupt stress), while responsive initiation (e.g., in response to a recurring or “anticipated” stress, like nutrient limitation) is in blue.

Inactivation, also referred to as growth arrest or the initiation of dormancy, has been well studied for several pathogens and model microorganisms, especially those that exhibit readily identifiable phenotypic states of deep dormancy, such as endospores, exospores, akinetes, cysts, and myxospores (e.g., reference 9). There are several categorical reasons for microbes to deactivate, and, within these categories, some underlying deactivation mechanisms may share commonalities (Fig. 2). One well-studied reason for cells to initiate dormancy is resource limitation (e.g., as summarized in reference 42), including carbon and other nutrients. Another is to respond to stressors unrelated to resources, such as intense or abrupt shifts in the physical or chemical environment, including temperature, moisture, osmolarity, and pH. Microbial interactions may also drive deactivation, including both intra- and inter-population engagements, suggesting that both kin- and non-kin interactions can be important in determining dormancy. For example, quorum-sensing has been shown to regulate persister formation in *Legionella pneumophila* (43). Pathogen persistence to antibiotics also shows how intense intermicrobial interference competition, which is competition facilitated by direct warfare (44), can lead to deactivation and dormancy. Interference competition typically results from limitations on shared resources, such as nutrients (e.g., reference 34) or space (e.g., in biofilms [45]). Finally, there are stochastic initiations into dormancy, in which a subpopulation enters dormancy as a bet-hedging strategy (e.g., references 46, 47).

Among these causes of deactivation, a period of starvation may be more likely to trigger entry into a deeper, molecularly regulated, and protective dormant state. On the other hand, a stress that is routine in the dynamic environment (e.g., seasonal or diurnal fluctuations in moisture, temperature, or oxygen availability) may be hypothesized to more likely result in a state that is more readily reactivated. This has been suggested by rewetting data from soil microbiomes, in which soils with a greater fraction of more active bacteria were more quickly responsive (17), but more work is needed to explore the ecological consequences of environmental stability for microbiome responses relative to their activity. The implications of environmental stability for seed banks (the dormant partition of the community, originally defined for plant communities) have been discussed thoroughly, especially with an evolutionary lens (48, 49). The idea is that populations that have been exposed to past stress exposure can adapt by evolving responsive regulatory mechanisms (e.g., two-component systems [50]). In cases of severe or novel stresses, the cell may assume a dysregulated inactive state because it has no specifically evolved response (e.g., reference 38) and then prioritize survival and repair of compromised functions. More cross-systems research is needed to identify and understand generalities in these processes.

Especially within the context of antibiotic persisters, several reviews exist on their cellular mechanisms of initiation (e.g., references 21, 40, 41), which are related to both the type of antibiotic exposure and the environmental context [21]). Here, we highlight a few recent studies that point to broader mechanisms underlying deactivation. For example, one common deactivation strategy is to interfere with ribosomal function and thus protein translation (as reviewed in reference 42). One mechanism for this is hibernation factors, which bind to ribosomes to inhibit new protein synthesis while preserving ribosome integrity (a strategy of “molecular hibernation”) (51). A hibernation factor (“Balon”) has recently been discovered in a psychrophile and found to be broadly phylogenetically distributed among bacteria and commonly detected in various environments (52). As another related strategy, RNA-binding proteins can inhibit or modify post-transcriptional regulation, and recent work has shown that the RNA-bound proteomes of vegetative and sporulated *Bacillus subtilis* cells are distinct, with the spore proteome containing, among other expected sporulation proteins, stress-indicative proteins like RNA chaperones (53). In addition, recent work has shown that low ATP availability in dormant cells is associated with protein aggregation, which may protect proteins from complete breakdown and thus allow the cells to repair and reuse them during reactivation (30). Finally, epigenetic regulation, such as DNA methylation, has been hypothesized to provide bacteria with a mechanism that encourages subpopulations to more responsively enter states of persistence given exposure to stress like antibiotics (54). If supported, epigenetic modification would add another deterministic regulation of bacterial dormancy and could provide discrete molecular signatures to probe dormancy in mixed populations and communities.

### The state of dormancy

Microbial dormancy is not often a complete cellular shut-down, but rather a dynamic state of reduced metabolism. Even the hardiest dormant states, such as endospores and exospores, can exhibit measurable cellular respiration (e.g., reference 55), albeit at lower levels than observed in vegetative cells. While dormant cells are thought to be protected from stress, they can die or lose viability, rendering them unable to reactivate and thus become evolutionary dead ends (56).

Dormant states can be responsive or “regulated” from a cellular perspective (e.g., reference 57), for example, when the cell employs a regulated response (Fig. 2) to a routine, gradually amplifying or low-intensity stress and, in some cases, arrests growth (57). In these states, molecular hibernation, the formation of a phenotypic resting stage (e.g., spore/cyst), halting of DNA replication and synthesis, and downregulation of transcription and translation are common cellular responses. For example, some starved *E. coli* dormant cells classified as VBNC can reduce their cell volume and cytosol content relative to exponential cells and maintain membrane integrity, while other VBNC cells lost cytosol completely and could not be reactivated despite intact membranes; these observations suggested a regulated entry into a protective physiological state that can respond to available nutrients as long as cytosol was maintained (27). Other work similarly proposes that the extent of reduced internal mobility from physical limitations on molecular reactions may correspond to different reactivity levels when conditions become favorable (58), and further delineate a deep versus shallow dormant state.

Dormant states could also be reactive and “dysregulated,” for example, when the stress is abrupt, novel, or severe (Fig. 2). In reacting to a stress, upregulation of DNA repair, the SOS response, and membrane repair are common cellular responses. For example, protein homeostasis (proteostasis) may also be compromised during some dormant states, and the cell may respond by employing strategies to rebalance (59, 60). Therefore, the ecological and environmental context will determine the characteristics of the dormant state.

### Reactivation

The reactivation from dormancy and resumption of activity are as varied as the mechanisms for dormancy initiation. As for initiation, reactivation can be deterministic, such as responding to an external molecular signal or cue (e.g., as in the case of resuscitation-promoting factors among Actinomycetota, e.g., references 20, 61, 62), newly available resources (e.g., as in the case of L-alanine for sporulated *Bacillus* spp., e.g., reference 63), or a release from stress. Reactivation could also be stochastic (e.g., references 64–66). While there remain large knowledge gaps about the biology of reactivation for most environmental bacteria, accounting for stochasticity in awakening among mixed-species microbial communities may provide important information for predicting a community’s capacity and pace of recovery after perturbation.

There is a theory that whether a cell has adopted a deterministic or stochastic response is related to the degree of temporal environmental heterogeneity in its habitat. In this framework, a relatively stable environment (one that is either typically very positive or very negative for the cell) favors deterministic processes, while relatively unstable environments favor stochasticity (e.g., reference 67). Benefits of stochastic switching include low to no investment in environmental sensing and thus the potential for subpopulations to respond rapidly by directing resources efficiently toward growth when the environment becomes favorable. However, reactivating in an unfavorable environment more likely results in cell death, leading to a depletion of the dormant pool with each attempt at awakening. Because reactivation may require a time lag for the cell to prepare for growth by utilizing newly available resources or repairing damaged cellular components before growing, there is also interest in understanding trade-offs associated with this time lag for the efficacy of stochastic versus deterministic switching (47).

Reactivating cells can acquire new ATP, disaggregate any proteins to restore proteostasis (e.g., references 30, 59, 68), ready ribosomes for translation by removing binding proteins and other hibernation factors (e.g., reference 27), and prepare for growth by shifting their gene regulatory profile (2). In some cases, specific metabolite signaling or release of metabolite-regulated inhibitors has been implicated in reactivation. For example, the release of metabolites that inhibit access to stored cellular glycogen enables reactivation in cyanobacteria (29). Hardier, deep-dormant phenotypes can have multiple layers of regulation for reactivation, typically mirroring the similarly complex processes of dormancy initiation. For example, the model spore former *Bacillus subtilis* maintains receptors on the spore membrane that are responsive to amino acids (such as L-alanine), which have been linked to cation release from the spore core, a signal for germination (69). Other related work suggests that dynamic electrochemical signals are also responsible for spore germination in bacilli (70). Finally, phage can induce reactivation from dormancy, showing that biotic interactions can also play a role (71).

## ECOLOGICAL CONSEQUENCES OF THE MICROBIAL ACTIVITY SPECTRUM

Ecological theory has traditionally treated dormancy as a binary state: an organism is either inactive or active, and this assumption has shaped how microbial contributions to ecosystem processes are conceptualized. However, the recognition that environmental microorganisms occupy a continuum of activity levels necessitates a reframing of ecological consequences through the lens of the activity spectrum. Importantly, many ecological outcomes attributed to “dormancy” emerge not from complete inactivity but from heterogeneity in activity levels within and among taxa, and from dynamic switching along this spectrum in response to environmental change.

There remain many ecological unknowns in environmental microbiology that cannot be addressed without quantifying activity (Box 1). These include understanding fundamental questions pertaining to biodiversity and the longevity and viability of cells, and predicting microbial functional stability after disturbances. We discuss a few of these in more detail here, emphasizing those concepts that draw on or could be enriched by considering an activity spectrum.

Box 1. **Ecological unknowns about the environmental microbial activity spectrum**

- **Biodiversity**: What is the diversity of the microbial dormant pool (i.e., “seed bank”) in environmental ecosystems? How compositionally similar is the dormant pool across the spectrum of activity levels, and as compared to the pool of dividing cells?
- **Longevity and viability**: How old is the deep dormant pool, and how does its age vary across environments? How long can environmental bacteria remain in a non-dividing state and remain viable? What is the relationship between the likelihood of cell death relative to its activity level?
- **Origins**: What part of the inactive microbial pool originates locally, what part is the outcome of dispersal from elsewhere, and does this differ depending on activity level? How can the sinks and sources of deeply inactive environmental microbes inform the movement of organisms or genes?
- **Temporal drivers**: How does the dormant pool change in its composition and depth of (in)activity over time and in response to disturbance? What is the potential of inactive populations to ensure stable functionality by increasing activity after disturbance?
- **Biotic interactions**: What are the consequences of varying levels of immigrant cell activities for the outcomes of coalescence? What mobile genetic elements are maintained by inactive cells?
- **Prediction and forecasting**: What are the contributions of activity switching to community-level microbiome dynamics? How can these be parameterized into forecasting models to understand microbiome response to short- or long-term stressors?
- **Memory**: How do the previous experiences of an inactive cell contribute to its ability to respond to present stimuli after it reactivates? How does epigenetics play a role in the ecological outcomes of deactivation and reactivation?

Notably, interpretations of ecological patterns can change when analyses focus on the active fraction rather than the DNA-based “total” microbiome profiles that include inactive, non-viable, and dead cells (e.g. references 3, 72–74). For example, only a handful of taxa were transcriptionally active in the rhizosphere of a drought-stressed legume, suggesting host selection from the thousands detected by DNA-based profiling (75). Likewise, removing the relic DNA strengthened some trends in soil beta diversity (73). These examples highlight why resolving microbial activity is important not only for interpreting ecological patterns but also for applying relevant frameworks.

One relevant ecological framework for considering how microbial functions respond to disturbance is the insurance hypothesis (76), which posits that biodiversity buffers ecosystem function in fluctuating environments through response heterogeneity among taxa. When viewed through an activity-spectrum lens, insurance may arise not only from taxonomic diversity but also from heterogeneity in activity levels across taxa, such that subsets of the community are poised to respond at different rates and intensities following disturbance. Populations occupying shallow versus deep dormant states may differ markedly in their responsiveness, lag times, and energetic costs of reactivation, thereby spreading functional contributions over time and stabilizing ecosystem-level processes, but more empirical data from environmental populations are needed to fully understand these differences. In cases of extreme disturbances, the most deeply dormant cells offer preservation, allowing for replacement of the disturbance-sensitive taxa and their functions afterward.

A related concept, functional redundancy, refers to the extent to which multiple microbial taxa are capable of performing the same function, and can be quantified as the number of unique functions detected as distributed across the taxa detected within the community (77–80). It is generally accepted that there is some degree of concordance between microbial community structure (which taxa are there and in what relative contributions) and functional outputs, although the strength of this relationship varies and depends on factors such as phylogenetic distribution. (81–83). Functional redundancy is typically quantified from DNA-based metagenomic profiles that cannot distinguish the signals from active, inactive, and dead community members (e.g., reference 84). As a result, conventional estimates implicitly assume that all detected taxa are equally capable of contributing to function. However, when microbial communities contain large fractions of low-activity and inactive cells, redundancy inferred from DNA-based community composition may overestimate functional stability, particularly over short ecological timescales. Equivalency not only in the function but also in its response rate and intensity becomes important to support redundancy.

Recent work has shown that, after an intense heating disturbance, the active partition of a soil bacterial community recovered more slowly than the total. The latter accumulated inactive taxa, contributing to perceived overall community resilience as far as recovery of taxonomic diversity, but could not have contributed to overall functional resilience due to their inactivity (85). Thus, from an activity-spectrum perspective, reactivation can be viewed as a time-delayed mechanism of functional rescue (86), whose effectiveness depends on the depth, viability, and composition of the dormant pool.

To what extent the dormant partition can safeguard active functions against environmental fluctuation is unknown, but this should depend on several factors. One key factor is the compositional coherence between the local active and dormant partitions. Although dormancy is often assumed to arise from *local* microbial growth followed by inactivation, this need not be the case. Dispersal connects communities across landscapes, as described by metacommunity theory (87), which considers dispersal alongside other assembly processes in shaping local community outcomes (e.g., references 88, 89). While microbes are considered readily dispersed due to their small size (90, 91), dispersal capabilities vary among taxa (e.g., reference 92). Sporulated (and therefore dormant) bacilli, for example, disperse extremely effectively and have been used as environmental tracers (e.g., reference 93). Thus, the protection afforded by dormancy is not necessarily independent of dispersal success. The environmental dormant pool, therefore, includes both locally contributed inactive cells and inactive cells that have been dispersed from elsewhere (85, 94). In principle, locally contributed dormant cells may be better adapted to the historical range of environmental variability, while the dispersed dormant cells may be less so. Conversely, immigrants may introduce traits that improve resilience to novel or extreme disturbances. The relative contributions of local versus immigrant sources to the dormant pool, and their consequences for routine versus novel perturbation, remain unresolved.

Dispersal does not introduce uniformly active immigrants, and arriving cells may also span an activity continuum from deeply dormant spores to metabolically active but non-dividing cells, with implications for immediate interactions and delayed community reassembly and clear consequences for community coalescence. Coalescence is the merging of two microbial communities from different origins, as is common at interfaces between habitats (e.g., references 95–97). Coalescence theory considers the biotic interactions that result from the merger and their roles in driving the assembly of the merged community. Previous work has shown that both reactivation with dispersal together can support greater soil community resilience than either factor alone (98). Because immigrants cannot interact until they activate, microbiome surveys that do not resolve activity levels may miss latent biotic interactions that emerge only after environmental change or time delays, which can then interrupt established biotic interactions. It remains unclear whether more active immigrants are more successful invaders than less active ones, which has implications for outcomes of coalescence.

Competition among active microorganisms can contribute to community assembly and impact functions. The Storage Effect (99–101) provides a framework for species coexistence that is maintained by temporal partitioning of activation. Two populations that have overlap in their niches can avoid direct competition by thriving at different times, with each maintaining a “storage” of population that is less active or inactive while its competitor flourishes. The Storage Effect allows for the avoidance of competition facilitated by the complementarity of competitor activity levels.

Quantifying and then accounting for heterogeneity in activity levels within environmental consortia (i.e., moving from a binary to a spectrum view) could reshape our understanding of microbial ecological phenomena. Although resolving activity could reduce noise from inactive community members (and clarify patterns in beta diversity), it will also reveal new, more complex signals that vary within and among taxa. For example, high within-taxon activity heterogeneity may reflect stochastic bet-hedging, whereas homogeneity may indicate strong deterministic control of activity shifts. Across taxa within a community, divergent activity levels may point to lineage-specific responses to local conditions, whereas uniformly low activity may indicate broad environmental limitation.

Another factor that could contribute to the dormant pool’s ability to maintain functional stability is its longevity and viability. The evolutionary consequences of a dormant genetic pool that can persist for generations have been well discussed elsewhere (e.g., references 7, 48, 102). Briefly, a more durable dormant pool allows populations to access a repertoire of genetic mutations that may be selected in a changed environment after reactivation. This contributes to the maintenance of genetic and genomic biodiversity within microbial communities, which may, over time, accumulate functional complementarity that enables them to withstand infrequent or novel disturbances. Thus, it is possible that the more durable a population’s dormant partition, the more genetic potential accumulates that can contend with a larger range of environmental fluctuations. Their accumulated genetic potential could also include maintained mobile genetic elements that may be transferred among the community when reactivated, with consequences for functions. However, there may be a trade-off between the longevity of the dormant partition (e.g., chronological life span [7]) and the viability of dormant cells.

Moving forward, many classical ecological concepts, like insurance, redundancy, coexistence, and resilience, may require reinterpretation when microbial communities are viewed as dynamically structured along an activity spectrum rather than partitioned into active and inactive states.

## DIFFERENTIATING MICROBIAL ACTIVITY STATES AND LEVELS

There is much recent interest in understanding the inactive and low-activity components of environmental microbiomes (3, 48, 103, 104). However, there are limitations in the availability of and access to technologies that distinguish activity states and levels in environmental populations and communities, which have impeded progress.

There are several challenges to quantifying environmental bacterial dormancy and activity. First, most bacteria lack obvious phenotypes that indicate an inactive state. Rather, there is evidence that some inactive bacteria reduce their cellular volume, rearrange but maintain their membranes, or otherwise modify their physiology without apparent outwardly drastic phenotypic changes (e.g. as reviewed in 1, 55). These metabolic and physiological differences between active and dormant cells are often detected using relatively lower-throughput methods, often at the single-cell level (105). Thus, there is a wide gap between the precise measurements that can be obtained from single cells in the laboratory and the need to quantify environmental microbial activity at population- and community-levels. Another challenge is the expectation of population-level heterogeneity in activity levels, as has been observed, for example, within biofilms (e.g., references 106, 107). Yet another challenge is a technical one: it remains difficult to obtain *in situ* activity measurements of environmental microbiota, as many methods rely on cellular labeling, incubations, or other assays that are necessarily removed from the environment. Given that transcript turnover is rapid, and can be within a few minutes (e.g., as reviewed in reference 108), even a brief lag between the moment of sampling and the moment of assay can skew some measurements. Finally, there is also a challenge of standardizing across assays and activity measurements so that different populations and communities from different ecosystems and contexts can be compared directly. Microorganisms that have different life-history strategies (e.g., oligotrophs vs. copiotrophs) may have fundamentally different ranges and limits for their activities (e.g., ribosomal gene copies relative to growth rates and efficiencies as per reference 109). Similarly, we may also expect that microbes that live in different environments, both in terms of physical-chemical characteristics and resource availability (e.g., an open ocean vs. eutrophic coastal water, equatorial vs. polar), may also exhibit different ranges in their activities. Thus, care must be taken to ensure that the most generalizable methods are used for research questions that assess environmental microbiome activity.

Here, we provide an overview of the major methods for detecting and discriminating activity among environmental microbial populations and communities (Table 1), starting with methods that detect universal activities of viable cells, and then moving to methods that capture activities from the highest to lowest relative levels along the activity spectrum. The general approaches represented by these methods can be divided into three categories: traditional microbiological methods, macromolecule assessment, and substrate or label uptake (Table 1, Box 2). The general strengths and limitations of each approach are discussed in Box 2, while technique-specific features and challenges are discussed by method below. We conclude with a description of promising emerging methods for activity assessment.

**TABLE 1:** Overview of the major methods used to determine activity levels or states in environmental microbial communities and populations (e.g., multispecies communities with heterogeneous activity states and levels)^*a*^

Activity targeted	Method	Binary or spectrum?	Category	Targets a cell or population?	How does it work?	References
Universal indicators of activity	Cell redox staining (CTC- RSG)	Spectrum	Traditional microbiological method	Cell	Cells are stained with a fluorogenic redox indicator dye that yields fluorescence when modified by reductases that are part of electron transport systems.	(110, 111)
	Bulk (heterotrophic) respiration	Spectrum	Traditional microbiological method	Population	Bulk or basal respiration is monitored with specific sensors and probes, detecting respiration-produced gases (CO_2_, CH_4_, NO_2_ are the most commonly monitored).	(112)
	ATP expenditure	Spectrum	Traditional microbiological method	Population	ATP is quantified with luminescence kits or biochemical assays.	(113)
Replication (DNA synthesis)	BrdU (halopyrimidine 5-bromo-3-deoxyuridine)	Binary	Substrate-uptake assays	Cell or population	Cells are incubated with alternative thymidine, which is incorporated during DNA synthesis. This labeled DNA is immunocaptured to distinguish it from unlabeled DNA.	(114)
	DNA-SIP: stable isotope probing	Binary	Macromolecule assessment	Population	Isotope-labeled substrates are taken up by cells and integrated into their DNA. Labeled (heavy) versus unlabeled DNA can then be fractionated with ultracentrifugation and analyzed with omic strategies to determine the active taxa.	(115)
Transcription (mRNA)	Metatranscriptomics	Binary	Macromolecule assessment	Population	RNA is extracted from the cell, reverse transcribed to cDNA, and then sequenced.	(116)
	RNA-SIP	Binary	Macromolecule assessment	Population	Same general method as DNA-SIP, but instead the RNA is targeted from unlabeled and labeled portions.	(117)
	FISH-TAMB: fluorescent *in situ* hybridization of transcript-annealing molecular beacons	Binary	Substrate-uptake assays	Cell	Based on the FISH strategy (explained below). This method relies on molecular beacons binding to intracellular mRNA. Labeled cells can grow and be later visualized using microscopy or sorted with microfluidics or flow cytometry.	(118)
Translation (rRNA/ribosomes)	16S rRNA to rRNA gene ratio	Binary	Macromolecule assessment	Population	RNA and DNA are co-extracted from a sample; the RNA is reverse-transcribed to cDNA; the 16S rRNA gene is amplified; and the RNA:DNA signal ratio is used to determine which populations are synthesizing ribosomes (e.g., active).	(119, 120)
	FISH: fluorescent *in situ* hybridization	Binary	Substrate-uptake assays	Cell	A specific probe with a fluorophore is hybridized to a cellular target. rRNA (or mRNA with FISH-TAMB) probes can target active cells.	(121, 122)
Metabolism (protein and metabolite biosynthesis)	Protein-SIP	Binary	Macromolecule assessment	Population	Isotope-labeled proteins are determined by mass spectrometry.	(123, 124)
	BONCAT: bioorthogonal non-canonical amino-acid tagging	Spectrum	Substrate-uptake assays	Cell	A modified amino acid is used as a substrate, synthesized into proteins of active cells, and a click-chemistry reaction is applied to bind a fluorophore to the modified amino acid.	(105)
	Proteomics/metabolomics	Binary	Macromolecule assessment	Population	All proteins synthesized in a system or in the environment are extracted, purified, then identified.	(125)
	Raman spectroscopy	Spectrum	Macromolecule assessment	Cell	Uses the scattering of monochromatic light to detect alteration of chemical bonds in the sample’s molecules, due to the laser interaction. Provides a detailed molecular composition, a “chemical fingerprint” of single cells, which can be paired with SIP or flow cytometry.	(126, 127)

^*a*^ The measurement of discrete variables would allow for binary differentiation of activity states (e.g., active vs. inactive/dormant), while continuous variables may allow for quantitative or semi-quantitative assessment of activity levels and thus could allow for assessment of the activity spectrum. Some methods that are typically used to determine a binary activity classification today could be enhanced in the future to gain spectrum-level insights. References provided are examples and not exhaustive.

Box 2. **General approaches used to determine environmental microbial activity**

- **Traditional microbiological methods** rely on straightforward observations of cellular phenotypes or broad metabolic capabilities, often using visual indicators of metabolic status. Three examples cited in this review are cell redox staining, bulk respiration, and ATP expenditure assays. These approaches are relatively easy to carry out because they use widely available materials, established protocols, and common laboratory equipment such as optical plate readers and microscopes. However, applying these methods to environmental microbiomes can be more challenging. Cells often need to be isolated from complex matrices or concentrated from dilute fluids before they can be analyzed, and environmental samples typically contain diverse microbial consortia, making it difficult to attribute observed activity to any particular taxon. Also, many of the traditional assays are not high-throughput.
- **Macromolecule assessment** includes today’s standard omics approaches that enable the characterization of the composition and identity of the major molecular components of RNA, DNA, proteins, and metabolites. These sequencing and profiling methods distinguish among molecular types and can be used to differentiate active and inactive microbial taxa or cells, and typically can be performed in high-throughput. A key limitation is that sequencing methods (e.g., metatranscriptomics, SIPs, proteomics) do not directly quantify and account for the community size (number of individual cells), leading to relativized or semi-quantitative data sets. Thus, these types of methods are often strengthened when paired with direct quantification.
- **Substrate-uptake assays** rely on adding a detectable substrate or label to a community (typically in a microcosm or mesocosm), incubating for a set time, and then distinguishing cells or populations that incorporate it from those that do not. These substrates and labels can include nucleotide probes (in the case of FISH), modified amino acids (BrdU or BONCAT), or isotope-labeled water, carbon, or nitrogen resources. Because these integrate into cellular macromolecules, they can provide highly sensitive measures of activity and are often considered the “gold standard” for identifying active cells. However, these approaches require experimentally defined incubation periods to optimize substrate uptake, meaning that observations are removed from the *in situ* conditions and may not fully reflect them. Practical constraints frequently necessitate incubation times that differ from biologically ideal time frames, for example, to ensure sufficient label incorporation for detection. Despite these limitations, these methods have yielded exceptionally precise insights into which microbes carry out specific activities.

### Universal indicators of activity

#### Redox and respiratory chain cell staining

Activity-indicating probes and dyes that target specific cellular processes are reliable methods that can be used to distinguish activity states, and, in some cases, microorganisms. The two commonly used stains for respiration activity are 5-cyano-2,3-ditolyl tetrazolium chloride (CTC) and RedoxSensor Green (RSG), though many other probes exist with diverse microbial and activity targets (128). CTC is reduced by bacterial cells under both aerobic and anaerobic conditions to formazan, a red, water-insoluble fluorescent product (110). RSG is modified by bacterial reductases to indicate cellular redox activity and produces green fluorescence (111, 128). Both stains allow detection of respiring cells using fluorescence microscopy or flow cytometry, and depending on the instrument, additional single-cell characteristics, such as activity intensity, cell size, and cell shape, can also be measured (129). Unlike CTC, RSG does not inhibit cellular metabolism, enabling the possibility of live-cell imaging (111). These stains can further support cell sorting across activity states (redox active or not) and levels (intensity of redox signal [111, 129]), allowing downstream analyses of omics-based characterization.

#### Metabolism indicators: microbial respiration rates and ATP expenditure

Microbial metabolic rates can also serve as indicators of activity states and levels. Here, we focus on two common measures: bulk microbial respiration and adenosine triphosphate (ATP) content. Bulk respiration (also called basal respiration) can be monitored continuously using deployed sensors or measured at single time points with portable field probes that detect carbon dioxide or other gases (e.g., methane, oxygen, nitrogenous gases). These measurements provide an *in situ* assessment of activity intensity. Carbon dioxide respiration reflects the collective activity of all heterotrophically respiring organisms. For soil samples, respiration is typically measured in the laboratory, where rates strongly depend on physicochemical conditions such as moisture, temperature, and aeration (112). For aquatic samples, oxygen consumption is often quantified using the Winkler method and paired with bacterial carbon production to estimate bacterial growth efficiency (130). Bulk respiration can also be used to estimate ATP expenditure, providing a proxy for microbial viability and activity (112).

ATP quantifications were originally used as proxies for total microbial biomass in environmental samples. It is based on luminescence and can be performed rapidly using commercially available kits across a wide range of sample types (113, 131). However, cellular ATP levels vary with growth stage, taxonomy, and cell size, all of which relate to activity. Thus, ATP levels can indicate activity intensities, but these intensities may vary across different taxa (a similar challenge to other activity assays when applied to mixed consortia). Across 100 water samples from diverse sources, including groundwater, drinking water, bottled water, river, lake, or wastewater effluent, the average microbial ATP content was 1.75 × 10^−10^ nmol per cell (113). These values align with estimates from (24), who reported ATP contents ranging from 0.2 to 7.1^−10^ µg per bacterial cell. In soils, an ATP concentration below 2 µg/g has been proposed as a threshold for inactive microbial communities, whereas higher values indicate active communities (24).

Both respiration and ATP measurements come with important considerations. Soil respiration measured in the laboratory may differ from field rates because handling and transportation disturb native conditions. Appropriate gas exchange and incubation times must also be selected based on soil texture and chemistry, which can complicate large-scale studies (112). Regardless, soil basal respiration remains a fast and reliable indicator of soil microbial activity and is valuable in broader activity-assessment frameworks (132). In aquatic samples, filtration steps used before respiration measurements can disrupt natural substrate availability and trophic interactions, potentially biasing metabolic estimates (130). Furthermore, ATP measurements can also vary depending on the kit or extraction method used, and the biases and optimization of this method for aquatic and soil samples have been discussed previously (24, 113, 131).

While basal respiration and ATP expenditure provide valuable functional insights, they are most informative when paired with complementary methods, such as staining to distinguish active and inactive cells or ‘omics approaches to identify the taxa underlying the measured activity. A recent work (132) advocates combining global soil respiration data sets with isotopic and biogeochemical measurements to generate new syntheses of microbial basal and potential activity, particularly in the context of climate-driven abiotic stress.

### Replicating cells: DNA-targeting methods

#### BrdU

BrdU relies on the cellular uptake of an analog of thymidine, the halopyrimidine 5-bromo-3-deoxyuridine (BrdU), during DNA synthesis by actively replicating cells (114, 133). It has been employed to distinguish replicating from non-replicating taxa across diverse environments (134, 135). BrdU-labeled DNA can be selectively immunocaptured, separating it from unlabeled DNA and thereby reducing interference from abundant, non-replicating taxa in complex communities (136). The labeled and unlabeled fractions can then be analyzed using standard molecular approaches like metagenome or amplicon sequencing and quantitative PCR (136). Because actively growing cells often represent only a small fraction of the community, BrdU labeling can produce low amounts of labeled DNA, making contamination and detection sensitivity notable challenges (114). BrdU can be paired with microscopy or flow cytometry to quantify replicating cells or can be used to sort replicating from non-replicating cells for downstream ‘omics. BrU-labeled taxa allow for a binary discrimination of recent replication activity (yes or no) and do not well differentiate activity intensities.

#### Stable isotope probing of DNA

Stable isotope probing (SIP) allows for the identification of microorganisms that actively assimilate specific substrates enriched with stable isotopes (137). By supplying microbial populations or communities with “heavy” isotope-labeled compounds (e.g., ¹³C, ¹⁵N, ²H, ¹⁸O), researchers can track the incorporation of these isotopes into cellular macromolecules and metabolites (115, 138, 139). While the first applications of SIP focused on phospholipid fatty acids (24), the modern SIP toolkit encompasses a broad range of approaches that monitor isotope uptake into DNA, RNA, proteins, and metabolites, enabling highly resolved insights into active substrate users within their communities.

Among these, heavy water has been used as a universal “substrate” and thus a general activity marker that does not interfere with the natural substrate pool. Applying heavy water as vapor in equilibrium within the mesocosm has further permitted “quantitative SIP” (also called “qSIP,” a term introduced by Hungate et al. [140]) that can be linked to estimate microbial growth from changes in taxon abundances (141), and has been applied to track microbial activities in low-moisture conditions (142).

DNA-SIP targets microorganisms that incorporate heavy isotopes into newly synthesized DNA, indicating active DNA replication and likely cell division. After incubation with a labeled substrate, ultracentrifugation separates heavy (labeled, active) from light (unlabeled, inactive) DNA, which is then fractionated and sequenced (115, 143). By comparing labeled samples and unlabeled controls, researchers can determine which taxa used the substrate and replicated during the experiment and apply a binary definition of activity that distinguishes replicating from non-replicating cells. DNA-SIP directly links microbial identity to substrate use, does not require cultivation, and can yield semi-quantitative activity estimates when paired with qPCR (allowing for a spectrum definition). It has been used across a variety of environmental samples, including seawater, soils, and plant-associated microbiomes (144). However, DNA-SIP detects activity only for the provided substrate, may be affected by cross-feeding, and requires substantial isotope incorporation for clear detection (145). It is also less sensitive in detecting rare or slow-growing taxa, which need more time to incorporate the isotope. SIP involves multiple technical steps, such as fractionation and pooling, that require expertise to perform reliably and with consistency to allow for comparisons across fractions and samples. A notable drawback of DNA-SIP is that changes in DNA buoyant density can also result from shifts in community composition, particularly toward microorganisms that have differing GC content, making it difficult to conclusively attribute density differences to isotope incorporation alone. This can complicate interpretation, especially in diverse communities in which GC-rich taxa may naturally co-migrate with labeled DNA. The use of unlabeled incubations can help to reduce this concern by allowing comparison between the unlabeled and isotope incubations. Due to the need to fractionate each sample and its unlabeled control and then sequence each fraction to comprehensively analyze one replicate, experimental designs that apply SIP can become quite large when investigating multiple factors or changes over time.

### Transcribing cells: mRNA-targeting methods

#### Metatranscriptomics

Metagenomics, the untargeted sequencing of DNA from an environmental sample, provides information about the taxonomic composition and functional potential of the microbial community. These data encompass DNA from active and inactive cells and, if extracellular DNA is not removed, also dead cells. In contrast, metatranscriptomics involves untargeted sequencing of cDNA reverse transcribed from sample RNA and captures the transcriptionally active fraction of the community. Prior to reverse transcription, rRNA reads can be depleted to maximize observation of the mRNA for sequencing, and the transcripts indicate the functional genes expressed at the moment of sampling. Although mRNA abundance does not always correlate directly with cellular activity levels (146–148), metatranscriptomics is widely used to identify active taxa in a binary sense (transcribing or not) and to infer recent cellular processes based on transcripts of functional genes and pathways (116). However, because of the large variation in gene regulation across cells and taxa, it does not distinguish activity levels when applied to mixed communities. Metatranscriptomes are most powerful when paired with reference metagenomes or genomes from the same sample or environment, which enables mapping of transcripts to specific populations and supports interpretation of the functional pathways they are expressing. This technique is thus strongly dependent on the availability and quality of metagenome or genome databases.

Numerous optimizations have been developed to improve the detection of transcripts that reflect recent gene expression. These include mRNA enrichment strategies (74), depletion or removal of rRNA (149), analysis of total rRNA (150), and sequencing of total RNA (total RNA-Seq) (151). Still, metatranscriptomics faces several technical challenges, including the short-lived mRNA molecules (as explained in Moran et al. [74]), which are highly susceptible to degradation by RNases (152). Also, bacterial mRNAs lack poly(A)-tails, complicating their capture from RNA pools dominated by abundant rRNA (153). In many environments, host-derived RNA can be abundant enough to obscure microbial transcripts (154). As a final challenge, it is difficult to glean reportable information about the percent active cells and populations using metatranscriptomics, especially in high-diversity communities like soils that are more likely to be incomplete in metatranscriptome observational effort. Internal standards can help to quantify the nucleic acid abundances, but this information is still difficult to directly relate back to individual cells and population sizes, the fundamental accounting units of ecology.

#### RNA-SIP

The overall workflow for RNA-SIP is like that of DNA-SIP (117, 124). RNA-SIP is generally sensitive to cell physiological changes because the transcriptome responds more rapidly to changes in environmental conditions than the genome (155). Unlike DNA-based labeling, RNA-SIP can detect activity in slow-growing or non-replicating microorganisms (e.g., reference 156), making it valuable for studying taxa that remain metabolically active without undergoing cell division. Because mRNA is less stable than DNA and represents only a small fraction of the total RNA pool (<5%), mRNA-SIP typically requires RNA enrichment or pre-amplification to obtain sufficient material for sequencing. It is typically applied as a binary definition of activity (transcribing or not), but could be semiquantitative with qSIP approaches (though it has the same limitations as other RNA-targeted methods in mixed communities due to large differences in gene regulation).

### Translating cells: ribosome-targeting methods

#### rRNA:rRNA gene amplicon profiling

The 16S rRNA molecule is a structural component of the bacterial and archaeal small ribosomal subunit and is essential to protein synthesis. Sequencing of the 16S rRNA gene became the standard for taxonomic bacterial identification because its conserved regions enable broad primer design, while its hypervariable regions allow taxonomic resolution at the genus, species, and sometimes strain level (119, 157). Because 16S rRNA is produced through transcription and used during translation, its presence and abundance provide information on which bacterial and archaeal species exhibit transcriptional and translational activity in a sample (120), and inform a binary definition of activity.

The ratio of 16S rRNA to the rRNA gene can be used to distinguish active from inactive taxa and is most effective for examining shifts in activity states under different environmental or experimental conditions (119). However, heterogeneity in ribosome content and regulatory dynamics across taxa, as well as variation in 16S gene copy number, limits the use of 16S rRNA as a measure of activity intensity (120, 157). As such, 16S rRNA is best viewed as a coarse indicator of the average activity of a population within its community. In practice, DNA and RNA are co-extracted from the same cell lysate, and RNA is then reverse transcribed to generate cDNA for amplification and sequencing. Taxa with 16S rRNA:rRNA gene above a defined threshold (often >1) are classified as active, based on the assumption that more active taxa contain many more ribosomes than gene copies.

Like all RNA-based methods (including metatranscriptomics), the rRNA:rRNA gene approach requires careful sample collection so that the RNA profile reflects the moment of collection. This typically involves rapid, minimally disruptive sampling followed by flash-freezing or stabilization with preservatives (e.g., reference 158). The ratio method can also yield “phantom taxa” that are detected in rRNA sequences but absent from the rRNA gene data (e.g., reference 159). Whether these represent genuinely rare but disproportionately active microbes or methodological artifacts (e.g., insufficient sequencing depth, amplification bias, or sequencing error) remains under debate (160). Finally, some taxa may protect their ribosomes during activity decrease (e.g., by employing hibernation factors as previously discussed), and so the presence of ribosomes does not necessarily indicate translation, while the relative changes in ribosome abundances can inform as to dynamic investment in translation.

As with all DNA-targeted omics approaches, extracellular or relic DNA (from necromass) will be included in rRNA gene measurements (72). Several methods can reduce or remove extracellular DNA during sample preparation, including magnetic bead-based capture (161), chemical exclusion such as propidium monoazide (157), or DNase digestion of samples before cell lysis (162). This allows researchers to remove potential necromass and inactive taxa and focus on the likely active component. Finally, computational modeling suggested that the ratio method may yield a high rate of false negatives (163), highlighting the importance of cautious interpretation and complementary lines of evidence. When executed correctly, this method can inform comparisons of active taxa (a binary definition) across related samples. It cannot be used to assess differences in relative activity between taxa, and it is not acceptable to use relative changes in the rRNA (cDNA) as a proxy for changes in absolute activity or in taxon abundance. Instead, those taxa that meet the ratio threshold (more rRNA than rRNA genes) should be selected from the data set, and then their DNA-based amplicons (rRNA gene data) analyzed as standard protocol in amplicon sequencing (75, 119).

#### rRNA-FISH

rRNA-targeted fluorescence *in situ* hybridization (rRNA-FISH) uses fluorescently labeled oligonucleotide probes that hybridize to abundant ribosomal RNA within intact cells, allowing taxon-specific detection and visualization (122). Because probe binding scales with rRNA content, signal intensity often correlates with ribosome abundance and thus with recent cellular activity or growth potential. rRNA-FISH can be coupled with other activity-sensitive methods, enabling direct readouts of substrate assimilation, growth, or metabolic flux in identified cells. Examples include MicroAutoRadiography-FISH (MAR-FISH), which links phylogenetic identity to functional uptake of radiolabeled substrates, and Raman-FISH or FISH combined with nanoscale Secondary Ion Mass Spectrometry (FISH-nanoSIMS), which detects stable-isotope incorporation at single-cell resolution (121, 164).

Conceptually, rRNA-FISH reports on the presence and abundance of target rRNA molecules, which makes it sensitive to changes in translational capacity. This technique detects proxies of activity (ribosome load, potential for protein synthesis, recent substrate incorporation when combined with isotopic or radiolabel methods) rather than direct measurements of specific enzymatic activities. In other words, rRNA-FISH allows for binary detection of microbial activity and, with careful calibration or signal-amplification FISH variants such as Catalyzed Reporter Deposition (CARD-FISH), quantitative estimates of active cell counts (165). Advantages for environmental samples include the potential for single-cell resolution, taxon specificity, spatial visualization in complex matrices, and compatibility with other imaging and isotope-probing methods. On the other hand, limitations include variable probe coverage, differences in cell permeabilization and signal strength across taxa and environmental matrices, potential underestimation of low-ribosome-containing cells, and the need for rigorous total-cell counts combined with careful protocol standardization to ensure comparability and reproducibility.

### Metabolizing cells: protein and metabolite-targeting methods

#### Protein-SIP

Protein-SIP provides one of the most direct measures of specific microbial metabolic activities (123). Unlike DNA- or RNA-SIP, which rely on density-based fractionation, protein-SIP uses mass spectrometry and can detect much lower levels of isotope incorporation, approximately 1% isotope incorporation with ^13^C-labeled substrates compared with the ~20% typically required for nucleic-acid-based SIP due to gradient separation constraints (166). Incorporation thresholds vary across isotopes (e.g., ^15^N vs. ^13^C), depending on their expected contribution to amino acid composition. The major strength of protein-SIP is its highly sensitive, quantitative measurement of isotope-labeled peptides, allowing detailed physiological inference (124). However, successful peptide identification requires high-quality genome or metagenome references, which remain challenging to obtain for many environmental communities.

#### BONCAT-FACS-seq

Bioorthogonal Non-Canonical Amino Acid Tagging (BONCAT) tracks translational activity of cells that incorporate an analog of the amino acid methionine, most commonly L-homopropargylglycine (HPG), into newly synthesized proteins (167). This approach leverages aminoacyl tRNA synthetases’ natural substrate affinity, enabling efficient HPG incorporation with minimal genetic modification or viability loss at low (nM to µM) concentrations (167, 168). Active cells can then be visualized using a “click chemistry” reaction, in which HPG-containing proteins are covalently linked to a fluorescent probe through a copper-catalyzed azide-alkyne bond (167, 169). BONCAT labeling has been applied across a wide range of complex sample types, including freshwater and seawater, freshwater and marine sediments, soils, oral biofilm, and plant endophytes, demonstrating its versatility for detecting translationally active cells *in situ* (169–171).

BONCAT has been paired with flow cytometry, especially fluorescent-activated cell sorting (FACS), to differentiate viable cells (identified via viability dyes) from translationally active cells that have incorporated HPG (171). These sorted pools can be subsequently analyzed using ‘omics approaches of subjected cultivation and enrichment, if cell viability is preserved. Because it targets translationally active rather than replication, labeling does not require growth and, thus, incubation times can be as short as 2–4 hours (168, 171). However, incubation time must be carefully optimized because very short incubations may underrepresent slow-growing microorganisms, whereas extended incubations can compromise cell viability (170). BONCAT labeling cannot inherently distinguish activity from direct substrate assimilation or activity arising from metabolic cross-feeding, necessitating appropriate experimental controls (170).

#### Proteomics and metabolomics

Improved availability of high-quality metagenomic datasets and technical advances in mass spectrometry have expanded the use of proteomics and metaproteomics for *in situ* identification and quantification of proteins made by microbial communities (123, 172). Because enzymes and other proteins reflect synthesized gene products, metaproteomics can directly link microbial activity to specific functions within a community (124). It also provides insights into microbial interactions by detecting secreted proteins such as effectors and antimicrobial compounds (125).

Metabolomics is the broad analysis of metabolites produced within a system. It is now widely applied to assess metabolic activity in environmental contexts such as wastewater or soils (173). Profiling metabolites and proteins is useful for tracking products, evaluating process efficiency, and identifying metabolites tied to biogeochemical processes (173, 174). Protein extraction from diverse, even complex, samples has improved substantially, and a small amount of extractable protein does not necessarily indicate low activity potential. However, uncertainties remain, including the lag time between the exposure to an environmental stress and the responsive synthesis of proteins (24). Moreover, linking metabolic activities to specific taxa in a complex community is difficult without labeling approaches, which SIP can provide (124).

A recent meta-omic study of wastewater treatment samples demonstrated that while metaproteomics uniquely detected expressed metabolic pathways and enzymes, it identified fewer taxonomic units than DNA-based approaches (175). Overall, (meta)proteomics provides valuable functional insight into microbial ecology but often benefits from integration with complementary biomarkers such as substrate labeling.

### Signature phenotypes: dormant structures and behaviors

As previously mentioned, many microbes can adopt specialized morphologies or phenotypes depending on their activity state, allowing for a clear binary definition of active or dormant. These specialized cells, like spores and cysts, indicate reduced activity and occur in many bacteria, fungi, and microeukaryotes (9). These phenotypes typically display low to no detectable metabolic activity and are highly resistant to environmental stress that may otherwise cause cell lysis and death (8).

Microscopy has long been used to detect such dormant phenotypes through characteristic morphological features (e.g., malachite green for endospore staining). Cryo-electron microscopy of vitrified sections, which preserves cell structures and morphology, can also reveal the detailed features of dormant forms (9, 176). While these tools are effective for visualizing dormant cells within a laboratory culture, applying them to complex microbial communities can be substantially more difficult.

There are other microbial phenotypes associated with reduced activity, including persister cells (177) and VBNC cells (178). Unlike dormant morphotypes such as spores or cysts, persister and VBNC states are identified based on contextual behavior rather than distinctive morphology. Therefore, these phenotypes are less often used as dormancy indicators in environmental contexts of mixed communities.

### Emerging technologies and approaches for microbial activity discrimination

#### FISH-TAMB

FISH-TAMB (fluorescent *in situ* hybridization of transcript-annealing molecular beacons) is a relatively recent method that can allow for the labeling of intracellular mRNA targets in minimal time, and without the need for washing, fixatives, or surfactants, as recently introduced by (118). The method relies on hairpin oligonucleotide sequences (molecular beacons) that contain a fluorophore and a fluorescence quencher, which bind to specific archaeal and bacterial mRNAs. Thus, molecular beacons are more sensitive and have less background interference than the more typical linear probes. FISH-TAMB can capture active but unculturable and rare taxa, with labeled cells growing apparently without interference from the molecular beacon. Labeled cells can later be visualized under confocal or epifluorescence microscopy, sorted using microfluidics or flow cytometry devices, and collected for ‘omics analyses. FISH-TAMB has been applied in aquatic environments, so protocol optimization for other environments is likely necessary. When microbial functional gene targets are known, FISH-TAMB may offer a solution to capture their activity more quickly *in situ* and without the need for lengthy incubations.

#### RAMAN-SIP and other single-cell approaches

Raman spectroscopy is a method that uses inelastic scattering of monochromatic light to detect the vibrations and rotations of chemical bonds generated by the laser interaction with the sample’s molecules. In microbial ecology, the sample is typically a microbial cell surface. The signal obtained is defined as a Single-Cell Raman Spectrum, and it provides detailed information on the molecular composition (“fingerprint”) of individual cells (126). Raman is a rapid, label-free, and culture-independent technique, and can be combined with stable isotope probing (Raman-SIP) to analyze community composition and function (127). Raman-SIP is based on the incorporation of specific isotopes by the community members of a sample, leading to changes in vibration frequencies and isotope-induced peaks in the Raman spectrum. For example, deuterium water incorporation enables detection of active individuals in a microbial community, a technique known as “Raman-Deuterium Isotope Probing” (Raman-DIP) (179). Similarly, using heavy water incubation in combination with Raman or NanoSIMS single-cell methods allows for a quantification of the cell activity level that can be used to calculate the active proportion (36). Targeted cells can be identified and retained for analysis, sequenced, and even selected for enrichment culture by coupling Raman-SIP to single-cell sorting technologies, such as Raman Activated Cell Sorting (RACS) (180). However, the Raman technique is limited by a low-scattering cross-section, resulting in a weak signal. Therefore, microbial cells that auto-fluoresce may have Raman signal interference. Modifications to amplify the Raman signal or extend its acquisition time are available (181). A key research limitation is that many labs may not have access to the instrumentation required for Raman, with a few labs that also specialize in its methodological development advancing most of the research that uses it.

Though technically challenging, there remains much promise in the capacity of single-cell approaches to differentiate differently active cells and populations, and to precisely determine their activated functions (e.g., by metabolite analysis or gene expression). For example, throughput RACS of heavy-water labeled cells has been developed and applied to complex communities (182), using either microfluidics or optical tweezers to isolate Raman-active cells. A pipeline called RAGE-Seq differentiates and isolates Raman-activated cells to enable single-cell genome sequencing on them (183). RAGE-Seq has been applied to complex soil communities to gain metabolic and taxonomic insights into activated cells and uncovered that many rare lineages of soil taxa were nonetheless active, providing insights into their functional contributions.

In the context of environmental microbiology, there is a clear need to scale up individual cell measurements to infer their consequences for ecological processes (e.g., components of biogeochemical cycling). Though the throughput of single-cell approaches is continually improving (105), the sample size (e.g., number of individual cell measurements) needed to have well-powered experiments and adequate taxonomic representation (especially of the rare biosphere [184]) can be especially challenging for highly diverse communities, such as soils or sediments. Another challenge is how closely these single-cell measurements represent the *in situ* state, as cells must be removed from their environment to conduct the assay.

#### Aggresome quantification

A recent study reported that the more and larger the protein aggresomes a cell had, the deeper its dormancy depth, and the less likely it was to resuscitate (30). Looking forward, aggresome quantification or other related methods that capture the extent of protein aggregation, stability, and proteostasis at the population and community levels could be of interest to develop for environmental microbiomes.

## LITERATURE SYNTHESIS: BACTERIAL ACTIVITY COMPARED ACROSS ENVIRONMENTS AND METHODS

The amount and proportion of active microorganisms and populations in environmental microbial communities remain unclear, though this information is expected to provide critical ecological insights that can help to understand the dynamics and responses of microbial functions within their ecosystems. To get a handle on general trends of activity across major environments, we performed keyword searches on the Web of Science Core Collection between 18 April and 30 November 2025 to identify primary literature that applied one of a suite of methods to quantify the proportion of active cells or active populations. We included terrestrial (soil or sediment), aquatic (freshwater or marine), and wastewater treatment environments. We screened each manuscript for relevant activity information, and, if possible, calculated or estimated values from figures or tables when the proportions of active cells or populations were not directly reported (see the supplemental material). Thus, these results are summaries of a mix of reported values and estimations and should not be quantitatively interpreted but rather used to assess overall trends and to identify gaps.

**Fig 3:**
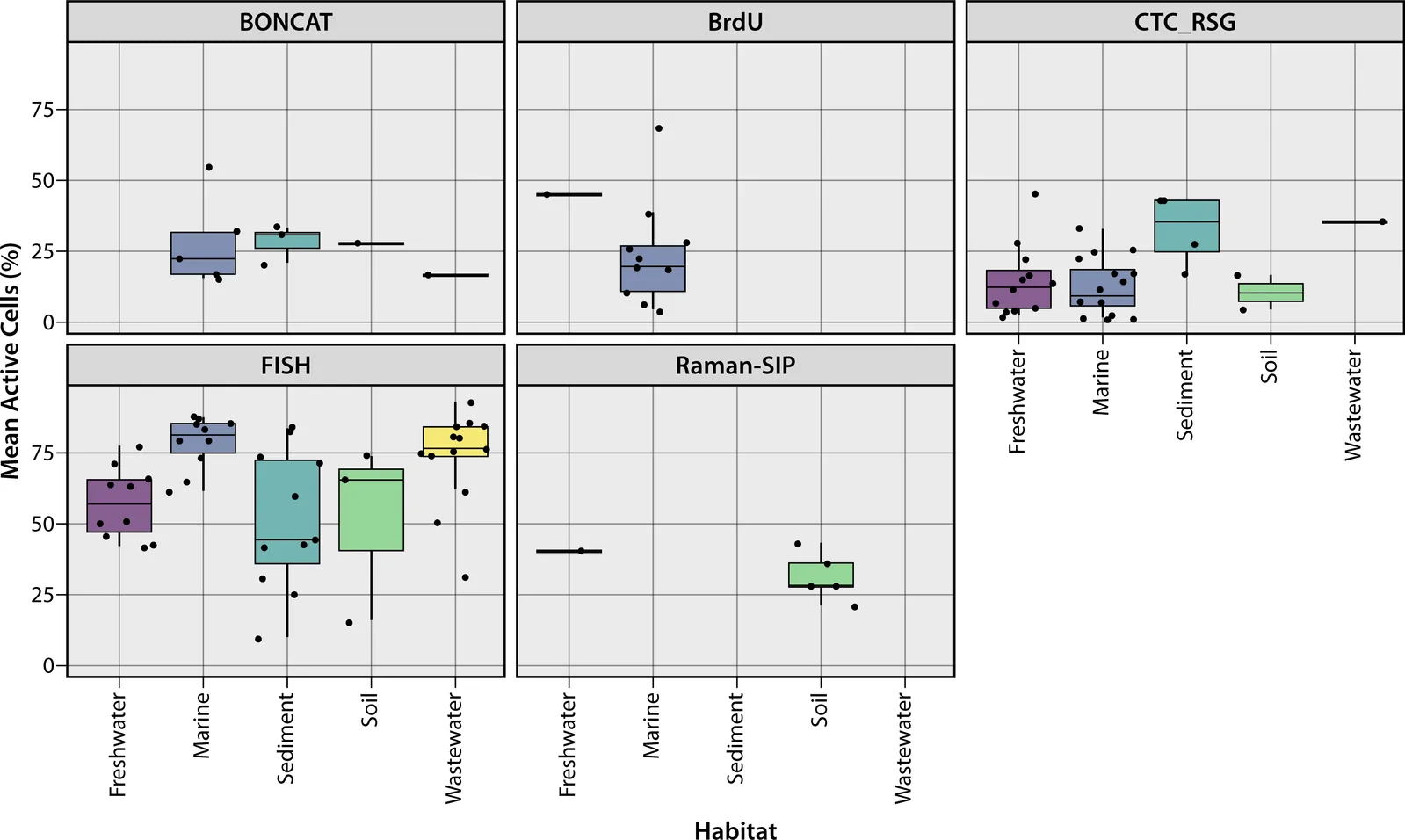
Mean percent active cells by method and habitat, summarized from the literature. The number of studies included in each category is indicated by the points overlaid on the boxplot. BONCAT is Bioorthogonal Non-Canonical Amino Acid Tagging with fluorescent-activated cell sorting. BrdU is a label-uptake method using a thymidine analog halopyrimidine 5-bromo-3-deoxyuridine. CTC_RSG are 5-cyano-2,3-ditolyl tetrazolium chloride and Redox Sensor Green, respectively, and are reductase staining methods that indicate redox-active cells. FISH is fluorescence *In Situ* hybridization, and here includes rRNA-FISH. Raman-SIP is a stable isotope probing with Raman spectrometry and here includes deuterium-labeled heavy water (a.k.a. Raman-DIP).

**Fig 4:**
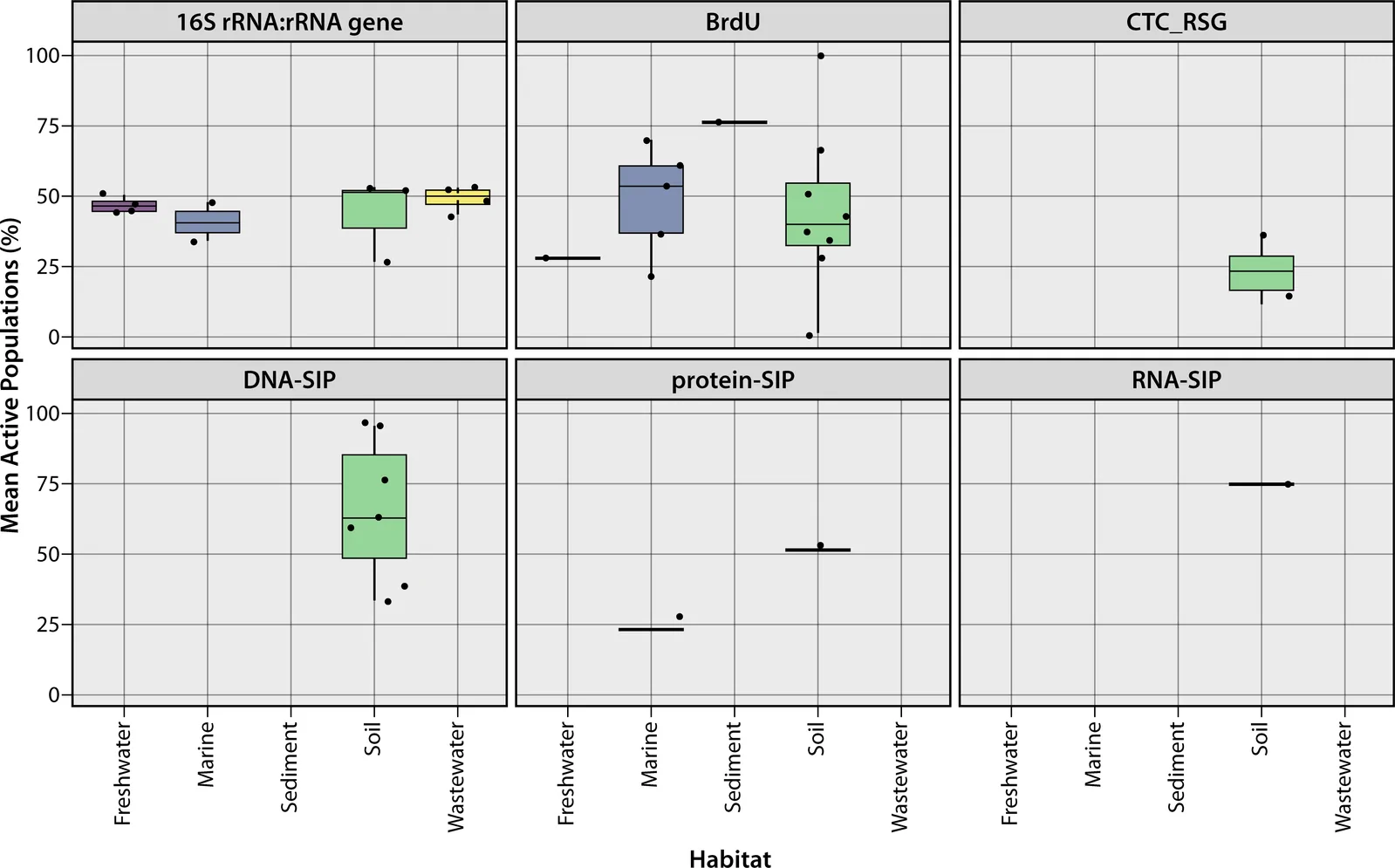
Mean percent active population by method and habitat, summarized from a literature analysis. The number of studies included in each category is indicated by the points overlaid on the boxplot. 16S rRNA:rRNA gene is a microbiome profiling method based on amplicon sequencing of cDNA from rRNA and the rRNA gene. BrdU is a label-uptake method using a thymidine analog halopyrimidine 5-bromo-3-deoxyuridine. SIP is a stable isotope probing, a substrate uptake method that can be used to distinguish cells with newly synthesized cellular DNA, protein, or RNA.

A first take-home point from the literature analysis is that, of 1,658 articles identified and screened, 90.8% of studies either did not report or did not provide the information needed to estimate the percent active cells or populations (Table 2). This suggests that the active proportion of a microbial community is not a result that researchers (or reviewers) consider relevant to their study’s core objectives or necessary to inform interpretation of other results. A second take-home point is that methods that quantified percent active cells (Fig. 3) were far more numerous than those that considered percent active populations (Fig. 4), despite their relatively lower throughput due to manual sample processing. Cell staining methods, such as respiratory chain staining with CTC or RSG, and the probe-based method FISH, were the most common. These methods have been applied across different ecosystems, with notable use in aquatic ecosystems. Among the few studies considering population-level activation, BrdU (combined with fingerprinting or sequencing-based microbiome profiling) and 16S rRNA:rRNA gene sequencing were the most common, and, in general, these have been applied broadly across different ecosystems.

**TABLE 2:** Summary of literature analysis, including the number of peer-reviewed articles screened, the number with relevant information regarding population or cell activity, and, among those studies included, the total number of samples from which discrete activity observations were available, summed across all of them^*a*^

Method	No. of screened articles	No. of included articles	No. of studied samples
CTC staining	94	33	569
RSG staining	3	2	18
BrdU	81	27	655
16S rRNA/rDNA ratio	67	13	579
DNA-SIP	546	7	31
RNA-SIP	359	1	75
Protein-SIP	89	2	33
FISH	187	51	439
BONCAT	132	10	401
RAMAN	100	6	95

^*a*^ CTC and RSG are 5-cyano-2,3-ditolyl tetrazolium chloride and Redox Sensor Green, respectively, and are reductase staining methods that indicate redox-active cells. BrdU is a label-uptake method using a thymidine analog halopyrimidine 5-bromo-3-deoxyuridine. 16S rRNA:rRNA gene is a microbiome profiling method based on amplicon sequencing of cDNA from rRNA and the rRNA gene. SIP is a stable isotope probing, a substrate uptake method that can be used to distinguish cells with newly synthesized cellular DNA, protein, or RNA. FISH is fluorescence *in situ* hybridization, and here includes rRNA-FISH. BONCAT is Bioorthogonal Non-Canonical Amino Acid Tagging with fluorescent-activated cell sorting. Raman is a stable isotope probing with Raman spectrometry and here includes deuterium-labeled heavy water (i.e., Raman-DIP).

A final, but perhaps the most important, observation is that the different methods applied do not necessarily agree in their relative rankings and differences in the percent active cells across habitats (Fig. 3). For example, though soils are consistently low across methods in their detected percent activity, they are not always the lowest reported, with some marine systems or sediment studies showing lower percent activity. This is different and more nuanced than what was summarized in 2011 from the selected activity-discriminating literature: that soils consistently had the lowest percent activity by a sizable margin as compared to freshwater, marine, activated sludge, human gut, and rumen environments (1). Our updated analysis suggests percent activity varies more within a given environment than previously appreciated, and that environments traditionally labeled as “very low” activity, such as bulk soils, may not be exceptional relative to others.

Direct, high-quality quantification of microbial communities (e.g., cell counts) and information about their active components (e.g., active cell counts or populations) is essential to advance microbiome research from an imprecise or semi-quantitative to a quantitative science. Strikingly, our review of the literature shows that although many studies that used activity-discriminating methods could report the ecological information of total cell counts (community size) and the number or proportion of active cells or populations, they rarely did. This suggests that our general understanding of active environmental cells and populations is limited in part due to underreporting, in addition to methodological challenges in technical capability, ease, and throughput. We recommend that reporting these data, which are fundamental units of ecological accounting (185), should be standard in microbiome research. These data are urgently needed, given the potential for stark changes in microbial activation and in population and community numbers with climate change and other anthropogenic disturbances.

## CONCLUSIONS: ADVANCING THE QUANTIFICATION OF ENVIRONMENTAL MICROBIAL ACTIVITY

Treating microbial activity as a quantifiable spectrum, rather than a binary state, is essential for mechanistic inference in environmental microbiology. This shift can address foundational knowledge gaps highlighted in Box 3, including understanding how much of the environmental microbiome is non-dividing but metabolically active and how these cells contribute to critical ecosystem processes, like respiration and biogeochemical transformations. Because it is expected that non-dividing cells dominate many environments, resolving their activity is critical for prediction and management.

Box 3. **Shifting perspective to a microbial activity spectrum: outstanding questions**

- **Generality**: Is there a universal definition of microbial activity that can be applied based on quantifiable, continuous metrics? What are the ranges of activity for environmental microbiomes, and how consistent are these within populations, communities, and phylogenetic lineages?
- **Importance and scale-up**: What are the collective contributions of non-dividing, low-activity cells to ecosystem processes? How can measurements scale up to capture community-level activity across heterogeneous cell activity states, and then be linked to environmental processes?
- **Cross-system synthesis**: Are there characteristic activity levels according to environment? How does this inform understanding of deterministic selection for microbial communities over space and time? Can we gain ecological insight with the standardization of activity ranges?
- **Harmonization for inference**: Do direct comparisons of different activity discriminating methods match the expectations according to relative importance of processes at different cell states (e.g., dividing, sporulated, metabolically active but non-dividing)? Are there conversion factors that would allow for comparisons across methods and studies?

Quantifying the active fraction can also change ecological inference in microbiome science, from the apparent strength of environmental drivers to conclusions about diversity, assembly, and stability. Progress, therefore, depends on pairing microbiome ‘omics data with basic ecological accounting of total community size, active cell abundance, and proportional activity, which we recommend should be reported routinely and transparently. This recommendation directly addresses cross-study comparability and harmonization challenges summarized in Box 3.

No single assay is universally “best” at discriminating activity. The most informative studies will instead integrate complementary approaches that target different cellular processes and, preferably, those processes that allow for a spectrum-based assessment between the states of dividing and deeply dormant, such as transcription, translation, and respiration. In parallel, we advocate training and investment in high-throughput, activity-resolving methods and broader access to specialized methods like Raman and SIP through shared facilities and pay-per-service models.

Until precise, throughput, activity-resolving approaches are more widely available, we recommend pairing reliable, accessible methods like direct cell counting (via microscopy or flow cytometry) with activity staining to quantify total and active cells and to anchor ‘omics interpretation. Although these methods can be low-throughput relative to ‘omics assays, their data can be operationalized across studies. There are options for increasing the throughput of these types of assays, for example, with automation or scaling for efficiency. As an example, our research team has optimized protocols for environmental cell redox staining and quantification with automated microscopy and image analysis (MATRIX [129]). The increased throughput afforded by this workflow has allowed us to assess the activity of complex soil communities, which we complement with ‘omics to understand how these microbiomes respond to our experimental treatments. We believe that the insights gained in investing in activity-discriminating approaches like MATRIX have been invaluable in advancing our objectives to understand microbiome resilience.

Ultimately, microbiome science will remain largely descriptive until we routinely measure who is active, how active they are, and how quickly they can change their activity state or level. Treating activity as a spectrum and pairing ‘omics with standardized ecological accounting of total and active cells will enable mechanistic tests of how communities respond to disturbance, from drought and warming to pollution and host-associated selection. Building and democratizing throughput, activity-resolving tools will therefore be pivotal for turning microbiome data into quantitative, predictive insight across environmental and human-impacted systems.

## SUPPLEMENTAL MATERIAL

Supplemental material mmbr.00354-25-s0001.docx Literature search terms and Table S1.

Tables S2 and S3 mmbr.00354-25-s0002.xlsx References returned by the literature search.

ASM does not own the copyrights to Supplemental Material that may be linked to, or accessed through, an article. The authors have granted ASM a non-exclusive, world-wide license to publish the Supplemental Material files. Please contact the corresponding author directly for reuse.
