# Supplementary material for: From dividing to dormant: embracing the full activity spectrum for environmental microorganisms
Source: Microbiol Mol Biol Rev. 2026 Jun 25;90(3):e00354-25. doi: 10.1128/mmbr.00354-25 (PMC13602961; doi:10.1128/mmbr.00354-25)
Supplement: Supplementary file 1 [file mmbr.00354-25-s0001.docx]

## Supplementary Material for

From dividing to dormant: embracing the full activity spectrum for environmental microorganisms

Ludivine Guigard^1^; Victor Bal^1^; A. Fina Bintarti^1^; Margaux Buron^1^; Emma Chavan^1^, Milena Gonzalo^1^; Xipeng Liu^1^; Wei Zheng^2^ and Ashley Shade^1^#

## Literature Analysis protocol and search terms for each method

This document provides the general literature search terms and keywords, as well as the specific research terms used for each activity-measuring method. For each method, the research date and the number of returned documents are provided. We excluded studies that focused on the activity levels of a single species, or a specific population and included studies that focused on environmental communities. Articles that did not use general probes (FISH), general substrates (SIPs), or general amplification primers (omics) were also excluded.

Supplementary Table S1: General parameters of our literature search

| **Parameters of literature search** | | | |
| --- | --- | --- | --- |
| **habitat** | **scale** | **study_type** | **organisms** |
| soil | cell | observational | bacteria |
| freshwater | population | experimental | archaea |
| marine |  |  | bacteria & archaea |
| sediment |  |  |  |
| activated sludge |  |  |  |

**General search terms and keywords:**

(((ALL=("microbial community" OR "microbial communities" OR microbiome OR microbiota OR bacteria* OR archaea* OR bacterioplankton))

AND ALL=(soil OR sediment OR marine OR freshwater OR aquatic OR “activated sludge”))

AND ALL=(**method specific)**

**Method specific terms, date of search and number of returned documents**

**CTC Staining:** performed on the 04/29/2025, 94 documents returned

((ALL=("microbial community" OR "microbial communities" OR microbiome OR microbiota OR bacteria* OR archaea* OR bacterioplankton)) AND ALL=(soil OR sediment OR marine OR freshwater OR aquatic OR “activated sludge”)) AND ALL=(“5-cyano-2 3-ditolyl tetrazolium chloride”)

**RSG Staining:** performed on the 04/29/2025, 3 documents returned

((ALL=("microbial community" OR "microbial communities" OR microbiome OR microbiota OR bacteria* OR archaea* OR bacterioplankton)) AND ALL=(soil OR sediment OR marine OR freshwater OR aquatic OR “activated sludge”)) AND ALL=(“redox sensor green”)

**16S rRNA:rDNA ratios:** performed on the 11/28/2025, 67 documents returned

((ALL=("microbial community" OR “microbial communities” OR microbiome OR microbiota OR bacteria* OR archaea* OR bacterioplankton)) AND ALL=(soil OR sediment OR marine OR freshwater OR aquatic OR “activated sludge”)) AND ALL=("rRNA/rRNA gene" OR "rRNA:rRNA gene" OR "rRNA:rDNA" OR "rRNA/rDNA" OR "16S ratio" OR "16S rRNA ratio" OR "16S ratios" OR "16S rRNA ratios" OR "16S rRNA and 16S rRNA gene")

**BrdU:** performed on the 04/18/2025, 81 documents returned

((ALL=("microbial community" OR “microbial communities” OR microbiome OR microbiota OR bacteria* OR archaea* OR bacterioplankton)) AND ALL=(soil OR sediment OR marine OR freshwater OR aquatic OR “activated sludge”)) AND ALL=(Brd-U OR BrdU OR bromodeoxyuridine)

We included studies that allowed for community-level assessment and excluded single-strain or population-focused manuscript that did not assess the total community.

**BONCAT:** performed on the 05/06/2025, 132 documents returned

(((ALL=("microbial community" OR "microbial communities" OR microbiome OR microbiota OR bacteria* OR archaea* OR bacterioplankton))

AND ALL=(soil OR sediment OR marine OR freshwater OR aquatic OR “activated sludge”))

AND ALL=("BONCAT-FACS-SEQ" OR "boncat facs seq" OR "boncat" OR "BONCAT-FACS" OR "bioorthogonal non-canonical amino acid tagging" OR "fluorescence activated cell sorting" OR "bioorthogonal noncanonical amino acid tagging"))

**FISH:** performed on the 04/29/2025, 187 documents returned

(((ALL=("microbial community" OR "microbial communities" OR microbiome OR microbiota OR bacteria* OR archaea* OR bacterioplankton)) AND ALL=(soil OR sediment OR marine OR freshwater OR aquatic OR “activated sludge”)) AND ALL=("in-situ hybridization" OR "fluorescence in situ hybridization")) AND ALL=("rRNA-targeted oligonucleotide" OR "MAR-FISH" OR "FISH-TAMB" OR "SIP-FISH" OR "Raman-FISH" OR "DOPE-FISH" OR "EL-FISH-NanoSIMS" OR "BONCAT-FISH")

We retained studies that explicitly used general bacterial or archaeal probes (e.g., EUB338 family, ARCH915) together with total-cell counting methods (DAPI) to report the proportion of probe-positive (putatively active) cells. Most exclusions were due to missing or incomplete data reporting, exclusive focus on narrow taxa without use of general probes, or absence of total-cell counts. Finally, the variation among protocols (e.g., use of EUB338 alone versus the EUB338 I+II+III mix, differing permeabilization/CARD methods, or differing thresholds for detection) likely contributes substantially to observed differences in proportions of active cells.

For all methods using stable isotope probing (SIP), we only include the studies that used H₂¹⁸O, ²H2O, or labeled complex substrates (e.g., plant residue, root litter) to minimize substrate bias while tracing activity of the whole microbial community.

**DNA-SIP:** performed on the 04/29/2025, 546 documents returned

((ALL=("microbial community" OR "microbial communities" OR microbiome OR microbiota OR bacteria* OR archaea* OR bacterioplankton)) AND ALL=(soil OR sediment OR marine OR freshwater OR aquatic OR "activated sludge")) AND ALL=(DNA-SIP OR DNA stable isotope probing)

546 documents returned total

**RNA-SIP:** performed on the 04/29/2025, 357 documents returned

((ALL=("microbial community" OR "microbial communities" OR microbiome OR microbiota OR bacteria* OR archaea* OR bacterioplankton)) AND ALL=(soil OR sediment OR marine OR freshwater OR aquatic OR "activated sludge")) AND ALL=(RNA-SIP OR RNA stable isotope probing)

**Protein-SIP:** performed on the 04/29/2025, 89 documents returned

((ALL=("microbial community" OR "microbial communities" OR microbiome OR microbiota OR bacteria* OR archaea* OR bacterioplankton)) AND ALL=(soil OR sediment OR marine OR freshwater OR aquatic OR “activated sludge”)) AND ALL=(Protein-SIP OR Protein stable isotope probing OR Protein-based stable isotope probing)

**Raman-SIP:** performed on the 10/27/2025, 100 documents returned

((ALL=("microbial community" OR “microbial communities” OR microbiome OR microbiota OR bacteria* OR archaea* OR bacterioplankton)) AND ALL=(soil OR sediment OR marine OR freshwater OR aquatic OR “activated sludge”)) AND ALL=( « Raman-SIP » OR Raman-Deuterium Isotope Probing » OR «

single-cell raman spectroscopy » OR «raman-activated cell sorting » OR « Raman-Activated Cell Ejection »)

We included studies on general communities analyzing bacteria or archaea in different environmental ecosystems but excluded any articles focusing on specific taxa or functions or which samples originated from human, animal or controlled culture. Articles non-including targeted values and method were also excluded from our analysis.
